# Downregulation of ABI2 expression by EBV-miR-BART13-3p induces epithelial-mesenchymal transition of nasopharyngeal carcinoma cells through upregulation of c-JUN/SLUG signaling

**DOI:** 10.18632/aging.102618

**Published:** 2020-01-06

**Authors:** Jing Huang, You Qin, Chensu Yang, Chao Wan, Xiaomeng Dai, Yajie Sun, Jingshu Meng, Yanwei Lu, Yan Li, Zhanjie Zhang, Bian Wu, Shuangbing Xu, Honglin Jin, Kunyu Yang

**Affiliations:** 1 Cancer Center, Union Hospital, Tongji Medical College, Huazhong University of Science and Technology, Wuhan 430022, China

**Keywords:** nasopharyngeal carcinoma, EBV-miR-BART13-3p, epithelial-mesenchymal transition (EMT), ABI2, SLUG

## Abstract

Existing evidence has shown that circulating Epstein-Barr virus (EBV)-miR-BART13-3p is highly expressed in plasma of nasopharyngeal carcinoma (NPC) patients, especially among patients with advanced diseases. However, the exact role that EBV-miR-BART13-3p plays in the development of NPC remains poorly understood. Here we show that up-regulated expression of EBV-miR-BART13-3p leads to increased capacity in migration and invasion of NPC cells *in vitro* and causes tumor metastasis *in vivo*. Furthermore, we find that EBV-miR-BART13-3p directly targets ABI2, known as a tumor suppressor and a cell migration inhibitor, drives epithelial-mesenchymal transition (EMT) by activating c-JUN/SLUG signaling pathway. Silencing ABI2 shows similar effects to overexpression of EBV-miR-BART13-3p, whereas reconstitution of ABI2 resulted in a phenotypic reversion, highlighting the role of ABI2 in EBV-miR-BART13-3p-driven metastasis in NPC. Besides, expression levels of ABI2 in NPC tissue samples correlate with N stages of NPC patients. Taken together, these results suggest a novel mechanism by which ABI2 downregulation by EBV-miR-BART13-3p promotes EMT and metastasis of NPC via upregulating c-JUN/SLUG signaling pathway.

## INTRODUCTION

Nasopharyngeal carcinoma (NPC), a type of malignant tumor originating from the nasopharynx epithelium, has an especially high incidence in Southern China and Southeast Asia [1]. The etiology of NPC is generally considered to be the result of a combination of genetic factors, Epstein-Barr virus (EBV) infection and environmental carcinogens [2]. Due to its high radiosensitivity and deep location, intensity-modulated radiotherapy (IMRT) remains as the mainstay of NPC treatment and achieves excellent loco-regional control [3]. However, most NPC patients are diagnosed at a loco-regionally advanced stage, with extensive local infiltration or neck lymph node metastasis [4]. Distant metastasis is the most common reason for treatment failure of NPC in the era of IMRT [4], while the molecular mechanisms of metastasis are still unclear.

EBV, a double-stranded DNA virus, was first isolated from lymphoblasts originated from Burkitt’s lymphoma samples in 1964 [5]. People around the world are generally susceptible to EBV, with more than 90% to 95% of adults becoming serologically positive [6]. EBV is associated with multiple human malignancies, including NPC, NK/T cell lymphoma and gastric cancer, *etc*. EBV infection has been found in almost all NPC patients, with the highest EBV titers in patients diagnosed with undifferentiated cancer [7]. EBV infection is closely related to the occurrence and development of NPC, while the pathogenesis is not completely clear yet. Studies have shown that NPC tumor express large amounts of miRNAs encoded by EBV, the amplest of which are encoded by BamH I-A rightward transcripts (BARTs) region of the viral genome, known as BART miRNAs [8]. BART miRNAs regulate both virus and cellular genes at transcriptional or post-transcriptional level by binding to their target mRNAs [9], leading to viral latency, immune evasion [10, 11], cell growth and proliferation [12, 13], apoptosis [14–16] and metastasis [17–20]. Thus, the functions of BART-miRNAs in NPC are complicated and multifaceted.

Increased plasma levels of EBV-miR-BART13-3p were found in newly diagnosed untreated NPC patients, and the elevated levels were more pronounced among those with advanced diseases [21]. We analyzed microRNA profiling data of human nasopharyngeal tissues (312 paraffin-embedded NPC specimens vs. 18 normal nasopharyngeal tissues) in GEO database, and we found that expression levels of EBV-miR-BART13-3p were higher in NPC tissues than in normal nasopharyngeal tissues. Moreover, expression levels of EBV-miR-BART13-3p were closely correlated with the extent of regional lymph node metastasis. Thus, we hypothesize that EBV-miR-BART13-3p may play an important role in the development of NPC.

In the present study, we showed that high expression of EBV-miR-BART13-3p enhances the migration and invasion of NPC cells *in vitro* and promotes tumor metastasis *in vivo*. The specific mechanism has been demonstrated that EBV-miR-BART13-3p directly targets tumor suppressor gene ABI2, which consequently up-regulates the c-JUN/SLUG signaling, eventually leading to epithelial-mesenchymal transformation (EMT) and tumor metastasis. Our findings confirm the role that EBV-miR-BART13-3p plays in tumor metastasis of NPC, which may provide new insights for clinical intervention strategies.

## RESULTS

### EBV-miR-BART13-3p promotes migration and invasion of NPC cells *in vitro* and propels tumor metastasis of NPC *in vivo*

We analyzed the non-coding RNA profiling data of human nasopharyngeal tissues (312 paraffin-embedded NPC specimens vs. 18 normal nasopharyngeal tissues) in GEO database (https://www.ncbi.nlm.nih.gov/geo/query/acc.cgi?acc=GSE32960) to investigate miRNA expression in NPC tissues. Several BART miRNAs were over-expressed in NPC samples, including BART10, BART7, BART4, BART13, BART16 and so on. Compared with non-cancerous nasopharyngeal (NP) samples, EBV-miR-BART13-3p was highly expressed in NPC samples (Supplementary Figure 1), indicating that EBV-miR-BART13-3p may play a crucial part in progression of NPC.

**Figure 1:**
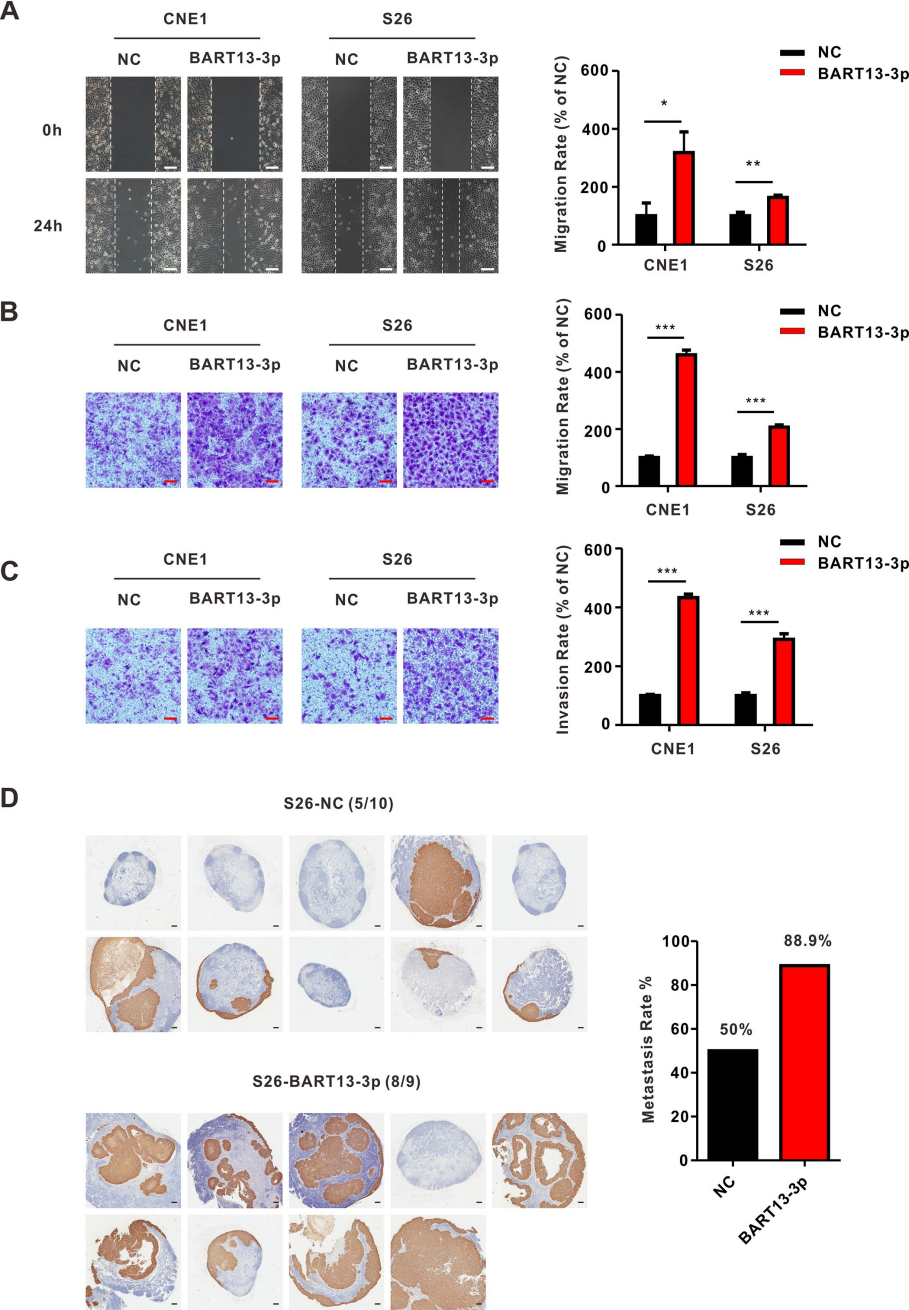
**EBV-miR-BART13-3p promotes migration and invasion of NPC cells *in vitro* and causes enhanced metastasis of NPC cells *in vivo*.** (**A**) Wound healing assay. Left panel: cell migration is measured by wound healing assay. CNE1 and S26 cells with BART13-3p overexpressed show higher migration capacity than negative control cells. Right panel: quantification of migration rate of CNE1 and S26 cells. Scale bar, 100μm. (**B**) Transwell migration assay. Left panel: up-regulated level of BART13-3p leads to increased migration of NPC cells using transwell assay without matrigel. Right panel: quantification of migration rate of NPC cells. Scale bar, 100μm. (**C**) Transwell invasion assay. Left panel: BART13-3p overexpression increases NPC cell invasion using transwell assay with matrigel. Right panel: quantification of invasion rate of NPC cells. Scale bar, 100μm. Error bars represent SEM. (**D**) S26-NC cells or S26-BART13-3p cells were inoculated under the right foot pads of the mice (n=8-10 per group). Popliteal lymph node metastasis on the same side were marked with immunohistochemical staining of pan-keratin. Scale bar, 200μm. (*P<0.05; **P<0.01; ***P<0.001).

To investigate the role of BART13-3p, we up-regulated the expression of BART13-3p in four EBV-negative NPC cell lines (CNE1, CNE2, S26 and 5-8F) by transient transduction (Supplementary Figure 2A). Wound-healing assay (Figure 1A and Supplementary Figure 2B) and transwell migration assay (Figure 1B and Supplementary Figure 2C) revealed that upregulation of BART13-3p significantly increased the NPC cell migration compared with negative control. Furthermore, transwell invasion assay with matrigel (Figure 1C and Supplementary Figure 2D) showed that upregulation of BART13-3p notably promote NPC cells invasion relative to negative control.

**Figure 2:**
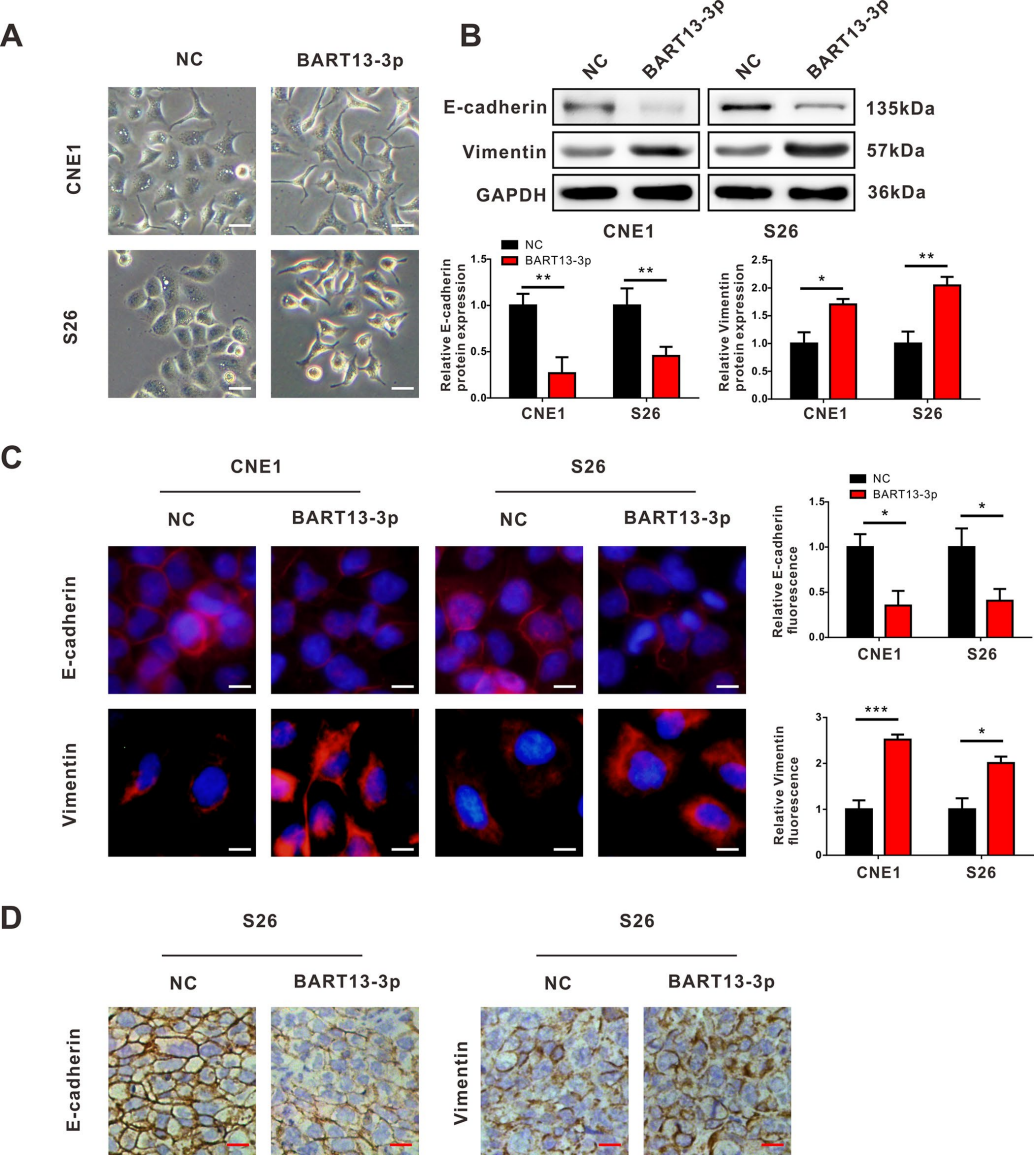
**EBV-miR-BART13-3p induces EMT of NPC cells.** (**A**) Cell morphological changes after transient transfection of BART13-3p in CNE1 and S26 cells. Scale bar, 100μm. Western blot (**B**), immunofluorescence (**C**) and immunohistochemistry (**D**) all showed that BART13-3p overexpression contributes to decrease of E-cadherin and increase of Vimentin in NPC cells or in NPC tumor specimens. Scale bar, 20μm (**C**) and 100μm (**D**). (*P<0.05; **P<0.01; ***P<0.001).

To make sure whether BART13-3p affected the invasiveness of NPC cells and tumor metastasis, the inguinal lymph node metastasis model was adopted. S26 cells stably over-expressed BART13-3p or negative control were established and injected into the right foot pads of nude mice. After five weeks of growth, the mice were killed. The ipsilateral inguinal lymph nodes were collected for *in vivo* imaging experiments, Hematoxylin and Eosin (H&E) staining and immunohistochemical staining. The primary foot pad tumors were obtained for immunohistochemical staining.

Whether judged by *in vivo* bioluminescence, H&E staining (Supplementary Figure 3) or positive expression of pan-cytokeratin (Figure 1D), the inguinal lymph node metastasis rate in BART13-3p group was higher than that of the control group.

Thus, these results suggest that EBV-miR-BART13-3p is over-expressed in NPC specimens. Upregulating the expression of EBV-miR-BART13-3p enhances NPC cell migration and invasion *in vitro* and promotes tumor metastasis of NPC *in vivo*.

### EBV-miR-BART13-3p induces EMT of NPC cells

We noticed that the morphology of NPC cells changed from an epithelioid form to a spindle, mesenchymal form when BART13-3p was over-expressed in CNE1 and S26 cells (Figure 2A), which suggested that EMT might be responsible for the effect of BART13-3p on NPC cell invasiveness. Therefore, we performed western blotting assay and immunofluorescence staining to detect the expression of epithelial marker (E-cadherin) and mesenchymal marker (Vimentin) in NPC cells with BART13-3p overexpression. Both western blotting and immunofluorescence staining showed that the upregulation of BART13-3p led to the decreased expression of E-cadherin and increased expression of Vimentin in CNE1 and S26 cells (Figure 2B and Figure 2C).

In addition, the immunohistochemistry assay showed the reduction of E-cadherin and the increased expression of Vimentin in BART13-3p stably over-expressed tumor tissues compared with control tumor tissues derived from the *in vivo* mouse models of NPC metastasis (Figure 2D). Consequently, EBV-miR-BART13-3p may enhance the invasiveness of NPC cells and promote tumor metastasis by inducing EMT.

### EBV-miR-BART13-3p directly targets cellular ABI2 in NPC cells

**Figure 3:**
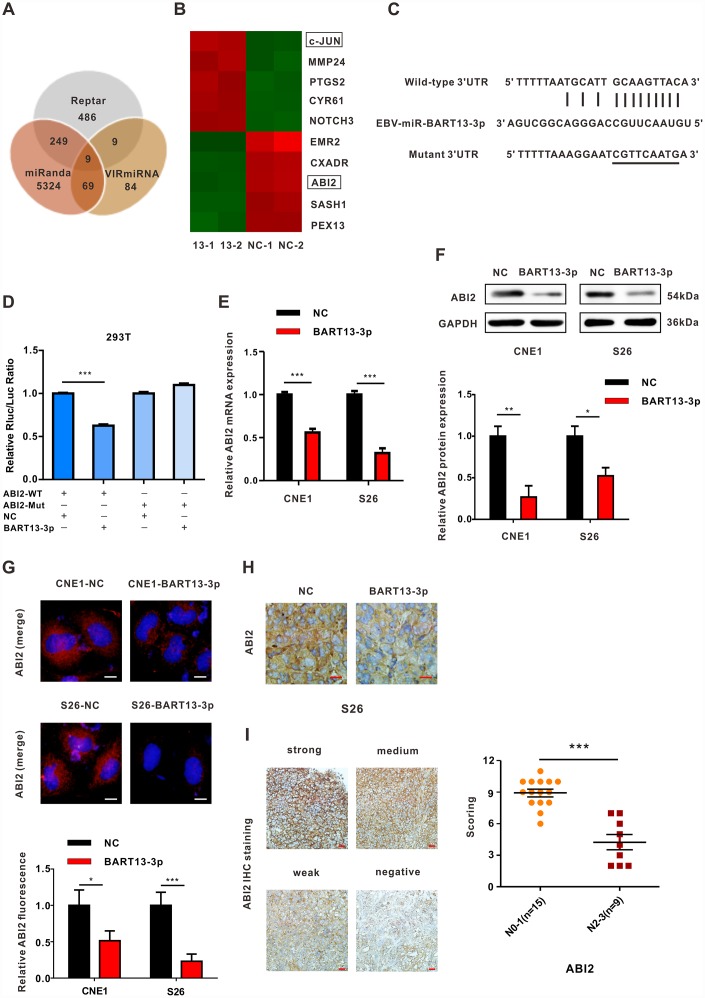
**EBV-miRNA-BART13-3p directly targets the cell migration inhibitor ABI2.** (**A**) Venn diagram of the number of target genes of BART13-3p predicted with three different databases—Reptar, miRanda and VIRmiRNA. Nine genes were predicted by all three databases, ABI2, TBC1D2B, ZNFX1, CSNK1G1, C9orf72, SLC41A1, NUFIP2, EIF5 and CCNE2. (**B**) Heat map obtained from mRNA microarray analysis (CNE1-BART13-3p versus CNE1-NC). CNE1 cells were transfected with BART13-3p mimics or NC for 24 h, and mRNA was isolated and then evaluated using microarray analysis. (**C**) Bioinformatics predictions showed the possible binding site of ABI2 3′-UTR region complementary with BART13-3p. Mutant sequences were showed as well. (**D**) Luciferase reporter assay. HEK293T cells were co-transfected with EBV-miR-BART13-3p NC or mimics and luciferase reporters carrying the predicted paired target site of ABI2 3′UTR (WT) or mutant (Mut). (**E**–**G**) RT-qPCR, western blot and immunofluorescence all indicated overexpression of BART13-3p contributes to decrease of ABI2 expression in NPC cells. (**H**) Immunohistochemistry on tumor slices of mouse models confirmed overexpression of BART13-3p reduced ABI2 expression in NPC tissues. (**I**) ABI2 protein expression in paraffin-embedded NPC specimens was detected by immunohistochemical staining. The staining intensity was divided into four grades, score ranging from 0 to 12. Scale bar, 20μm (**G**) and 100μm (**H**, **I**). Error bars represent SEM. (*P<0.05; **P<0.01; ***P<0.001).

In order to explore how BART13-3p promoted EMT and cell invasiveness enhancement, we used Reptar, VIRmiRNA and miRanda databases to predict the target gene of BART13-3p (Figure 3A). By taking the intersection of the target genes predicted by the three databases, we noticed a potential candidate gene, Abl interactor 2 (ABI2), which is known as a cell migration inhibitor and a tumor suppressor [22, 23]. Besides, ABI2 is also one of the differentially expressed genes identified by Agilent gene expression microarray (CNE1-BART13-3p vs. CNE1-NC, Oebiotech, Shanghai, China). Overexpression of BART13-3p in CNE1 contributed to a decrease in ABI2 mRNA expression (Figure 3B).

Further bioinformatics analysis showed that the 3′UTR region of ABI2 had a site complementary to the seed sequence of BART13-3p (Figure 3C). To confirm if BART13-3p could directly target ABI2, we conducted dual-luciferase reporter assay. Apparently, co-transfection with BART13-3p mimics significantly inhibited the luciferase activity of the wild-type 3′UTR of the ABI2 reporter gene. Whereas, the luciferase activity of the reporter gene was not affected by BART13-3p mimics anymore when the binding site of ABI2 3′UTR was mutated, indicating that BART13-3p could combine with the 3′UTR region of ABI2 (Figure 3D).

Subsequent quantitative real-time polymerase chain reaction (qRT-PCR) experiments revealed that overexpression of BART13-3p reduced the mRNA expression of ABI2 in CNE1 and S26 cells (Figure 3E). Similarly, western blotting and immunofluorescence staining confirmed that upregulation of BART13-3p decreased the protein expression of ABI2 in these two NPC cell lines (Figure 3F and Figure 3G). Immunohistochemical (IHC) staining on tumor slices of mouse models indicated that overexpression of BART13-3p significantly reduced the expression of ABI2 in S26-BART13-3p tumors relative to the control tumors (Figure 3H). Detection of the ABI2 protein by IHC staining showed that the protein expression of ABI2 in clinical NPC samples was remarkably reduced in N2-3 stages compared with N0-1 stages (Figure 3I).

Taken together, EBV-miR-BART13-3p directly targets the tumor suppressor ABI2 and reduces the expression of ABI2 *in vitro* and *in vivo*.

### Knockdown ABI2 causes similar effects on NPC cells to overexpression of EBV-miR-BART13-3p

**Figure 4:**
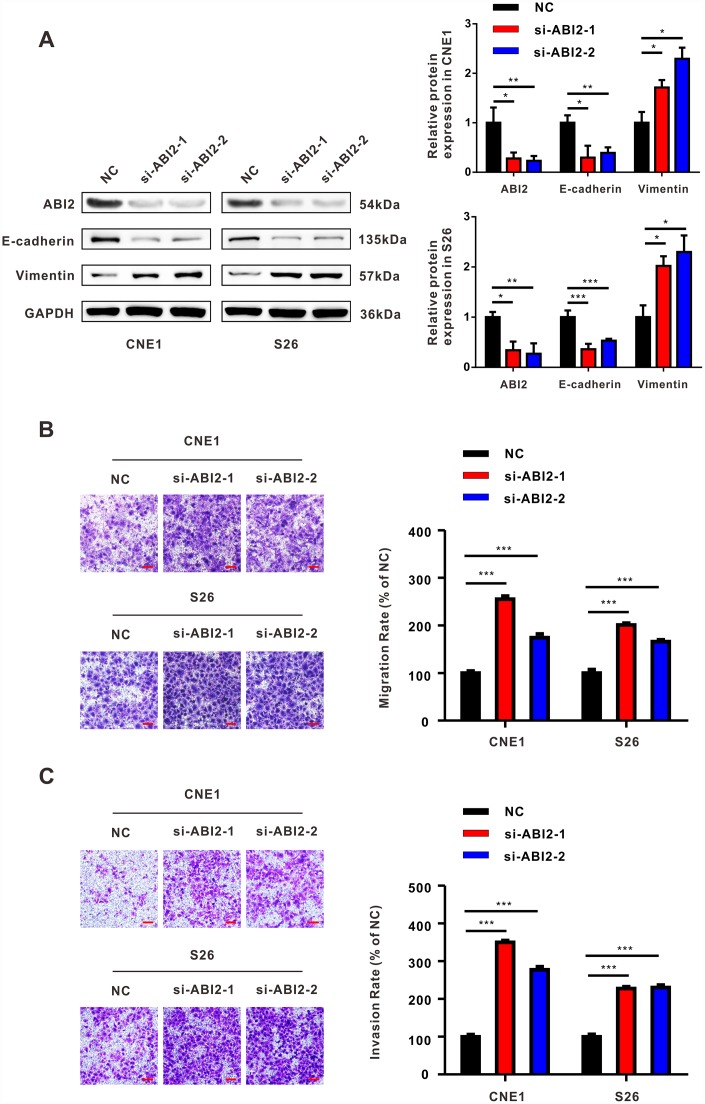
**The effect of down-regulated ABI2 on NPC cells is similar to that of overexpressed EBV-miRNA-BART13-3p.** (**A**) Western blot showed that knockdown of ABI2 by siRNA was effective. Downregulation of ABI2 caused decreased expression of E-cadherin and increased expression of Vimentin. (**B**, **C**) Transwell assay showed that siABI2 enhances migration and invasion of CNE1 and S26 cells. Right panel: quantification. Scale bar, 100μm. Error bars represent SEM. (*P<0.05; **P<0.01; ***P<0.001).

To explore whether ABI2 acted as a cell migration inhibitor in NPC, we used small interfering RNA (siRNA) technology to knock down ABI2 expression. Similar to BART13-3p, knockdown ABI2 contributed to the decreased expression of epithelial marker (E-cadherin) and increased expression of mesenchymal marker (Vimentin) in CNE1 and S26 cells (Figure 4A).

Besides, knockdown ABI2 promoted migration and invasion of both two NPC cells as revealed by transwell assay with or without matrigel (Figure 4B and Figure 4C). Collectively, knockdown ABI2 plays a similar role that EBV-miR-BART13-3p plays in EMT and NPC cell migration and invasion.

### Both EBV-miR-BART13-3p overexpression and ABI2 knockdown propels EMT through c-JUN/SLUG signaling

**Figure 5:**
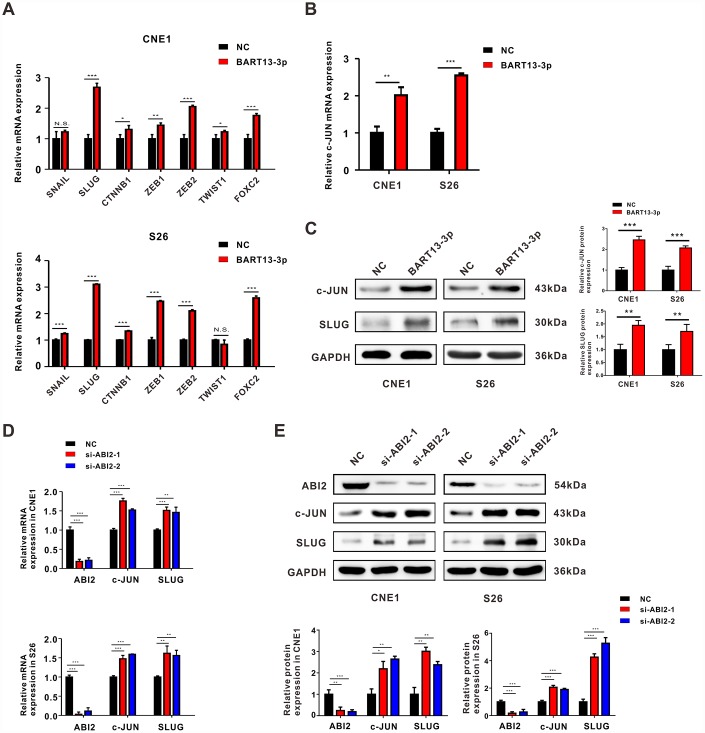
**Both EBV-miR-BART13-3p overexpression and ABI2 knockdown propels EMT through c-JUN/SLUG signaling.** (**A**) mRNA expression of common EMT-TFs regulators (SNAIL, SLUG, CTNNNB1, TWIST1, FOXC2, ZEB1 and ZEB2) were detected by RT-qPCR in CNE1 and S26 cells. (**B**) RT-qPCR assay showed the mRNA expression of c-JUN was up-regulated in NPC cells with high levels of BART13-3p. (**C**) Western blotting assay showed the protein expression of c-JUN and SLUG was up-regulated by BART13-3p. (**D**, **E**) Knockdown of ABI2 by siRNA increased both mRNA and protein expression of c-JUN and SLUG. Error bars represent SEM. (*P<0.05; **P<0.01; ***P<0.001; N.S. P>0.05).

In consideration of the pivotal roles that EMT-related transcription factors (EMT-TFs) play in the process of NPC metastasis, we examined the expression levels of some common EMT-TFs regulators (SNAIL, SLUG, CTNNNB1, TWIST1, FOXC2, ZEB1 and ZEB2) after up-regulating BART13-3p expression. Among all these EMT-TFs, SLUG exhibited the most significant changes in both CNE1 and S26 cell lines (Figure 5A). Meanwhile, we noticed that the mRNA expression level of c-JUN was increased in the differentially expressed genes screened out by Agilent gene expression microarray (Figure 3B). Several pieces of research have reported that c-JUN could bind to the promoter of SLUG, contributing to increased SLUG expression and induction of EMT [24, 25]. Thus, we performed qRT-PCR and western blotting assay to examine the expression of c-JUN and SLUG after overexpressing BART13-3p or knocking down ABI2. As expected, both mRNA and protein expression of c-JUN and SLUG was up-regulated (Figure 5B–5E).

Taken together, all these results suggest that BART13-3p induces EMT through activating c-JUN/SLUG signaling.

### Restitution of ABI2 rescues the phenotypes generated by EBV-miR-BART13-3p

As confirmed above, both over-expressed BART13-3p and under-expressed ABI2 can lead to EMT and NPC metastasis by inducing c-JUN/SLUG signaling. We were eager to clarify whether BART13-3p promoted cell invasion and migration by suppressing ABI2, therefore the following rescue experiments were carried out.

**Figure 6:**
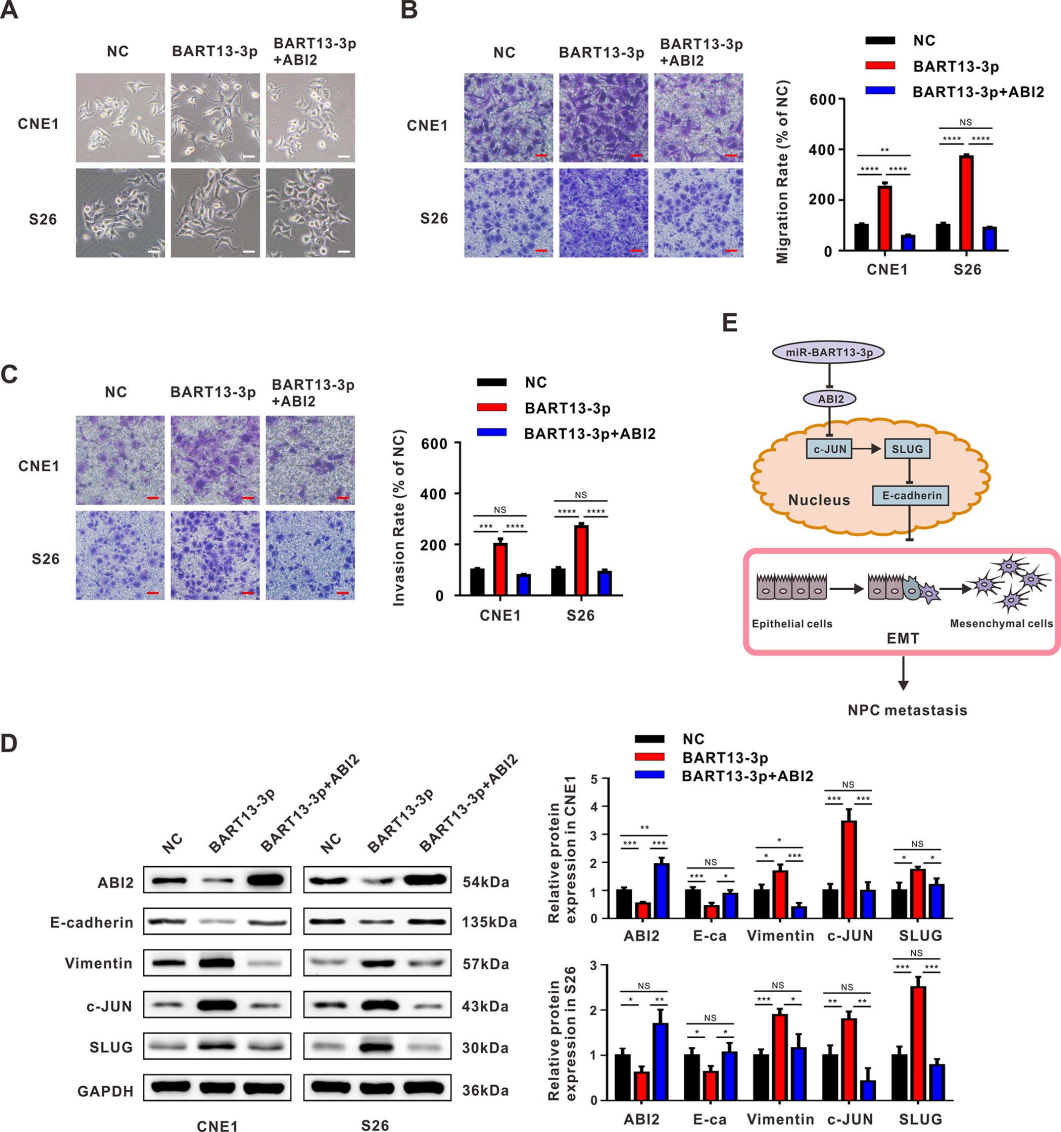
**Restitution of ABI2 rescues the phenotypes generated by EBV-miR-BART13-3p.** (**A**) Restoration of ABI2 reversed the morphological changes of NPC cells induced by BART13. (**B**) Restitution of ABI2 increased the protein expression of E-cadherin and reduced Vimentin expression in NPC cells with elevated level of BART13-3p measured by western blot. Meanwhile, c-JUN/SLUG signaling was also reversed. (**C**, **D**) Transwell migration assay and invasion assay validated that reconstitution of ABI2 reduced the cell migration and invasion of BART13-3p up-regulated NPC cells. (**E**) A schematic of BART13-3p inducing increased c-JUN/SLUG signaling and leading to EMT of NPC cells mediated by ABI2. Scale bar, 100μm. Error bars represent SEM. (*P<0.05; **P<0.01; ***P<0.001).

We transfected plasmid to overexpress ABI2 in CNE1 and S26 cells with elevated BART13-3p level. The restoration of ABI2 expression level reversed the effect of BART13-3p on the morphology of NPC cells (Figure 6A). Transwell migration assay and transwell invasion assay demonstrated that re-expression of ABI2 in CNE1-BART13-3p and S26-BART13-3p decreased cell migration and invasion compared with the relative ABI2-negative plasmid control (Figure 6B and Figure 6C). Furthermore, the protein expression levels of c-JUN and SLUG were significantly decreased after overexpression of ABI2 in BART13-3p over-expressed cells. The up-regulated protein expression of E-cadherin and down-regulated protein expression of Vimentin also indicated that the effect of BART13-3p inducing EMT was reversed by ABI2 recovery (Figure 6D).

Therefore, the enhanced NPC cell invasiveness and tumor metastasis potential resulted from EBV-miR-BART13-3p overexpression are dependent on ABI2 inhibition (Figure 6E).

## DISCUSSION

EBV infection is closely linked with the pathological process of NPC [26]. EBV-encoded miRNAs, especially BART-miRNAs, have decisive influences on this process [27]. BART-miRNAs are continuously and highly expressed in NPC cells [28]. Growing evidence suggests that BART-miRNAs can not only maintain the latent state of virus and mediate immune escape, but also correlate with the proliferation, apoptosis, invasion and metastasis of host cells. For instance, BART2 has an effect on the transition from latent to lytic viral replication by targeting BALF5 [10]. BART5 protects NPC cells from apoptosis by downregulating the expression of PUMA [14]. BART1 and BART7-3p induce NPC metastasis via suppressing the tumor suppressor PTEN [17, 18]. Therefore, the functions of BART-miRNAs in NPC are complicated and multifaceted.

It was reported that BART13-3p was highly expressed in the plasma specimens of patients newly diagnosed with NPC and correlated with their TNM stages [21]. By re-analyzing data in GEO database, we further confirmed that BART13-3p was highly expressed in NPC tissues compared with normal nasopharyngeal tissues, suggesting that BART13-3p may play a crucial part in the occurrence and development of NPC.

With transient transfection we found that overexpression of BART13-3p led to enhanced invasive and migration ability of NPC cells, which is consistent with previous findings.

We noticed that the cytological morphology of NPC cells underwent pseudopod-like and fusiform changes after overexpression of BART13-3p, suggesting that BART13-3p probably promoted migration and invasion of NPC cells through EMT. EMT is the key step of tumor metastasis. In the process of EMT, tumor epithelial cells lose cell polarity and cell adhesion ability, and are endowed with higher migration ability and invasiveness [29]. EMT is characterized by loss of epithelial markers and gain of mesenchymal markers [30]. Thus, we examined the expression of epithelial marker (E-cadherin) and mesenchymal marker (Vimentin). As expected, overexpression of BART13-3p led to increased expression of E-cadherin and decreased expression of Vimentin in NPC cells both *in vitro* and *in vivo*.

miRNAs function in mRNA silencing and translational inhibition through partially complementary pairing with the 3′ UTR regions of their target genes [31]. To identify potential target genes of BART13-3p, we used Reptar, VIRmiRNA and miRanda databases to predict target genes through the principle of complementary base pairing. Nine common target genes were predicted, while only ABI2 and SLC41A1 were differentially expressed between CNE1-BART13-3p and CNE1-NC detected with RNA-sequencing. We chose ABI2 for further study since ABI family members were reported to be regulators of cell motility [32–34]. Luciferase reporter assay further confirmed that ABI2 was indeed the target gene of BART13-3p and expressions of ABI2 mRNA and protein were inversely correlated with levels of BART13-3p in NPC cells *in vitro* and *in vivo*. Moreover, the expression of ABI2 in specimens from NPC patients were inversely related to their N stages.

Although previous studies have shown that ABI2 inhibits cell migration, they have not linked ABI2 to EMT. In our study, silencing ABI2 perfectly phenocopied the effect of overexpressing BART13-3p, contributing to EMT. To further explore the specific molecular mechanism, we quantified expressions of several common EMT-related transcription factors induced by BART13-3p overexpression and found SLUG expression increased most. The SNAIL superfamily, including SNAIL and SLUG, is a central group of EMT regulators [35]. At the molecular level, SLUG-mediated EMT is associated with its ability to transcriptionally repress the gene expression of epithelial marker E-cadherin, causing inhibition of cancer cell adhesion and promoting the migratory capacity [36]. Besides, Our RNA-sequencing results showed that the expression of c-JUN mRNA was elevated. c-JUN could combine with SLUG promoter, leading to an increase of SLUG expression and induction of EMT [24, 25]. Elevated c-JUN/SLUG signaling has been found in multiple cancer types and shows a significant association with invasion and metastasis [37, 38]. We further confirmed the up-regulation of c-JUN/SLUG after overexpression of BART13-3p or down-regulation of ABI2 at both mRNA and protein levels. Overexpression of ABI2 partially offset the effects of BART13-3p in our rescue experiments. To summarize, our study proposes that BART13-3p targets ABI2 to promote EMT of NPC cells through the c-JUN/SLUG signaling pathway. Our research elucidates the specific molecular mechanism of BART13-3p in promoting NPC invasion and metastasis and is the first one to report ABI2 in inhibiting EMT. However, it is not clear how ABI2 regulates this signaling pathway, thus further studies are warranted. Interestingly, Xu *et al*. recently reported that BART13 facilitated the proliferation and metastasis of NPC cells by targeting the NKIRAS2/ NF-κB signaling pathway [39]. It indicates that there are more than one pathway works in the process of BART13 facilitating metastasis.

A recent study showed that vesicle-bound BART13-3p in circulation is able to distinguish NPC from other head and neck cancers and asymptomatic EBV-infections, suggesting that BART13-3p has the potential to become a biomarker for diagnosis of NPC [40]. Since our research indicates that BART13-3p changes the biological behaviors of NPC cells and promotes their malignant characterization through c-JUN/SLUG signaling, targeting which may be a new therapeutic strategy for NPC. Therefore, BART13-3p can not only serve as a potential biomarker for the diagnosis of NPC, but also may become a molecular target for NPC treatment.

## MATERIALS AND METHODS

### Cell line and cell culture

Human NPC cell lines CNE1, S26, CNE2 and 5-8F were obtained from the Sun Yat-sen University Cancer Center (Guangzhou, China). The HEK293T cell line was purchased from the American Type Culture Collection (ATCC, Manassas, VA, USA). The NPC cells were cultured in RPMI-1640 medium (Gibco, Grand Island, NY, USA) supplemented with 10% fetal bovine serum (Gibco), 100U/ml penicillin and 100ug/ml streptomycin. The HEK293T cells were cultured in DMEM medium (Gibco, Grand Island, NY, USA) supplemented with 10% fetal bovine serum, 100U/ml penicillin and 100ug/ml streptomycin. All cell lines were maintained at 37°C with 5% CO_2_.

### Patients and tissue specimens

All NPC (not pretreated with radiotherapy or chemotherapy) specimens were collected and confirmed histologically in Union Hospital, Tongji Medical College, Huazhong University of Science and Technology, Wuhan, China. This study was approved by the Institutional Review Board of Huazhong University of Science and Technology. All experiments were performed with written informed consent. Twenty-four NPC specimens with TNM staging were used for immunohistochemical staining. The staging was performed according to the NPC-AJCC 8^th^ version. N staging indicates the extent of spread to regional lymph nodes, ranging from N0 to N3. Clinical staging is determined by combining the T, N and M classifications. All patient information is listed in Supplementary Table 1.

### Plasmid preparation and cell transfection

The expression vector pcDNA3.1 (http://designgene.com.cn/) containing the whole coding sequence of ABI2 and the control vector pcDNA3.1 were purchased from DesignGene (Shanghai, China). Plasmid DNAs were purified with TIANprep Mini Plasmid Kit (TIANGEN, China). Both CNE1 and S26 cells were transfected with 200ng plasmid DNA using Lipofectamine 2000 reagent (Invitrogen). Twenty-four hours after transfection, the cells were harvested for qRT–PCR and western blotting analyses. miRNA mimics or inhibitors (miRNA antisense oligonucleotides) were transfected at 50nmol/l with Lipofectamine 2000 reagent (Invitrogen). The EBV-miR-BART13-3p mimics (5′-UGUAACUUGCC AGGGACGGCUGA-3′) and associated negative control (5′-UUCUCCGAACGUGUCACGUTT-3′) were constructed by GenePharma (Shanghai, China).

### RNA interference

The sequences of small interfering RNA (siRNA) targeting ABI2 are as follows: si-ABI2-1, 5′-GGA ATTACGTTGAGTCTAT-3′; si-ABI2-2, 5′-CCACGTT CTTACTTGGAAA-3′. siRNAs were transfected by Lipofectamine RNAiMAX transfection reagent (Invitrogen, Camarillo, CA, USA). 48 hours later, cells were harvested and analyzed by real-time quantitative PCR and immunoblot analysis.

### Lentivirus production and infection

Lentiviral (GV281, Ubi-Luc-MCS-IRES-Puromycin) particles carrying EBV-miR-BART13-3p precursor and its control sequence were synthesized by GeneChem (Shanghai, China). S26 cells were infected with recombinant lentiviral transducing units plus 8mg/ml Polybrene (Sigma-Aldrich, St Louis, MO, USA) and selected with puromycin (1μg/ml) following the protocol from GeneChem. EBV-miR-BART13-3p expression was confirmed by qRT-PCR.

### Wound healing assay

Transfected CNE1 and S26 cells were seeded into a six-well plate, then the confluent cell monolayers were scratched with 200-ul pipet tips, washed with PBS to remove suspended cells, and cultured in serum-free media for 24h. Images were taken with an inverted microscope (Olympus, Japan).

### Transwell migration and invasion assay

For the transwell migration assay, NPC cells were resuspended in 200μl serum-free RPMI-1640 medium and then added to the upper chambers of 24-well transwell plates (6.5 mm insert, 8.0 μm pores, costar 3422). For the transwell invasion assay, cells in 200μl serum-free RPMI-1640 medium were seeded in Matrigel-coated transwell chambers. 500μl medium supplemented with 10% FBS was placed into the lower chamber. After incubation for 24h, the cells on the surface of the upper chamber were removed, and the migrated or invaded cells were fixed with 4% paraformaldehyde and stained with crystal violet. The stained cells were counted under an inverted microscope (Olympus, Japan).

### qRT-PCR

Total RNA was extracted with RNAiso plus reagent (TAKARA, Dalian, China) according to the manufacturer’s instructions and measured quantitatively by NanoDrop ND-1000. cDNA was synthesized with the ReverTra Ace qPCR RT Kit (TOYOBO, Osaka, Japan). The qRT-PCR was performed in triplicate with SYBR Premix ExTaq (TaKaRa, Dalian, China). The primers of the interested genes used for amplification are listed in Supplementary Table 2. RPU6B and GAPDH were used respectively for normalizing the expression of miRNA and mRNA. The fold changes were calculated using the comparative quantification (2 −ΔΔCt).

### Western blot

Cells were lysed in cold RIPA buffer containing protease inhibitor. Total proteins were separated by sodium dodecyl sulfate-polyacrylamide gel electrophoresis (SDS-PAGE) and then blotted onto the PVDF membrane (Millipore, Billerica, MA, USA). After being blocked with 5% skimmed-milk powder in Tris-buffered saline with Tween-20 (TBST), the membrane was incubated with the primary antibodies at 4 °C overnight, and then it was incubated with the secondary antibodies. The antigen-antibody complex on the membrane was visualized by ECL detection reagents (Beyotime Biotechnology, Shanghai, China). All antibodies are listed in Supplementary Table 3.

### Immunohistochemistry

Protein expression in tissues was determined by immunohistochemistry. In brief, tissue sections were blocked with goat serum after using citrate buffer to retrieve antigen, and then incubated with the primary antibodies at 4 °C overnight. After being incubated with horseradish-peroxidase (HRP)-conjugated secondary antibody, the tissue sections were reacted with 3,3-diaminobenzidine, counterstained with hematoxylin and dehydrated in graded ethanol. The protein expression was evaluated based on the percentage of the positive area and staining intensity. For statistical analysis, the intensity of immunostaining was scored as negative, weak, medium and strong, and the IHC scores were evaluated, ranging from 0 to 12.

### Immunofluorescence

NPC cells were cultured on coverslips overnight, washed twice with PBS for 5 min each time and fixed with 4% paraformaldehyde, then permeabilized with 0.5% Triton-X-100 in PBS for 15 min at 4°C. Subsequently, cells were blocked for nonspecific binding with 5% bovine serum albumin (BSA) in PBS for 30 min and incubated with primary monoclonal antibodies at 4°C overnight. The cells were then washed three times and incubated in the dark with secondary antibodies for 1 h at room temperature. After being counterstained with DAPI for 20 min, the slides were viewed under a fluorescence microscope (Olympus).

### miRNA target predictions

EBV-miR-BART13-3p candidate targets were obtained from Reptar, VIRmiRNA (http://crdd.osdd.net/servers/virmirna/) and miRanda databases. The miRanda programme was used to predict duplex complementary pairing between human ABI2 3′-UTR and EBV-miR-BART13-3p.

### Luciferase reporter assay

HEK293T cells (1.5×10^4^) were cultured in 96-well plates overnight and co-transfected with 50nM EBV-miR-BART7-3p mimic or NC, 200ng wild-type or mutant 3′UTR vector of ABI2. Forty-eight hours after transfection, the luciferase activities were analyzed with a Dual-Luciferase Reporter Assay System (Promega, Madison, WI, USA). Transfections were performed in triplicate and repeated in three independent experiments.

### *In vivo* xenograft tumor models

The BALB/c nude mice (4–5 weeks old, male) were purchased from Beijing Hua Fukang Bioscience Company (Beijing, China). Animal experiments were approved by the Animal Care and Use Ethics Committee of Tongji Medical College, Huazhong University of Science and Technology. In order to observe tumor metastasis, the inguinal lymph node metastasis model was performed. S26 cells (5×10^5^/25ul) that stably over-expressed luciferase/EBV-miR-BART13-3p or luciferase/NC were injected into the right foot pads of the mice (n=8-10 per group). After 5 weeks’ growth, the mice were killed. The foot pad tumors and inguinal lymph nodes were removed, detected *in vivo* bioluminescence, then fixed with 4% paraformaldehyde for H&E staining and immunohistochemistry.

### Statistical analysis

Each *in vitro* experiment was independently performed at least three times. Data were expressed as the mean ± SEM (standard error of the mean), unless otherwise indicated. Statistical significance was analyzed using Student’s t-test for two groups, one-way ANOVA (analysis of variance) analysis for multiple groups. P value < 0.05 was considered to be statistically significant.

## Supplementary Material

Supplementary Figures

Supplementary Tables
