# Supplementary material for: Downregulation of ABI2 expression by EBV-miR-BART13-3p induces epithelial-mesenchymal transition of nasopharyngeal carcinoma cells through upregulation of c-JUN/SLUG signaling
Source: Aging (Albany NY). 2020 Jan 6;12(1):340–58. doi: 10.18632/aging.102618 (PMC6977665; doi:10.18632/aging.102618)
Supplement: Supplementary Figures [file aging-12-102618-s002..pdf]

SUPPLEMENTARY FIGURES

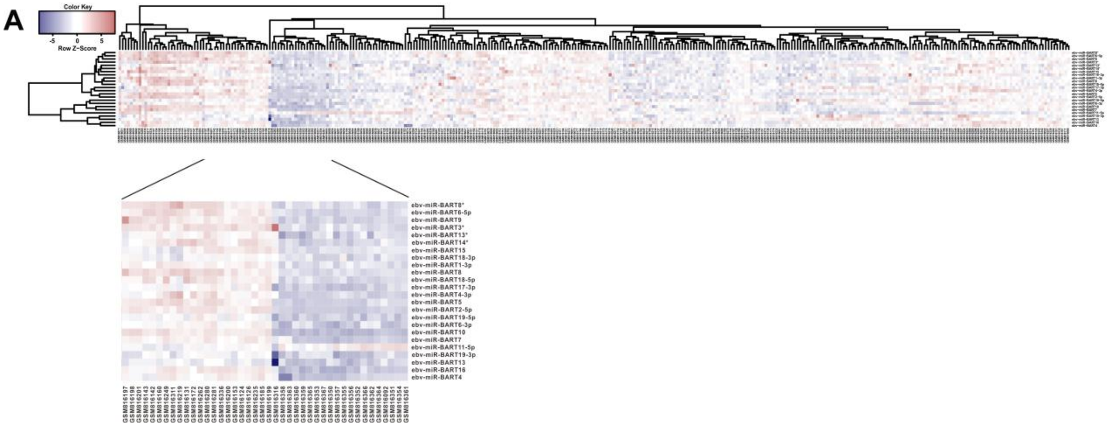

Supplementary Figure 1. (**A**) Heat map produced by re-analyzing the non-coding RNA profiling data of human nasopharyngeal tissues (312 paraffin-embedded NPC specimens vs. 18 normal nasopharyngeal tissues) in GEO database.

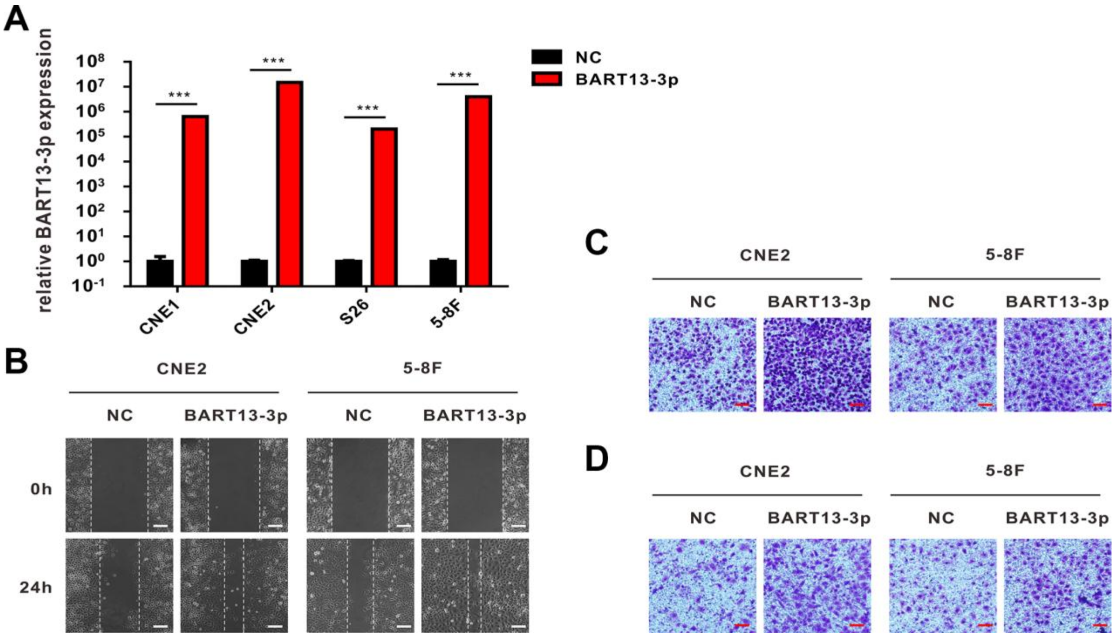

Supplementary Figure 2. EBV-miR-BART13-3p overexpression promoted migration and invasion of CNE2 and 5-8F cells. (**A**) CNE1, CNE2, S26 and 5-8F cells were transfected with BART13-3p mimics or negative control for 24 h and then harvested. Real-time PCR was used to determine the miRNA expression levels of BART13-3p. (**B**) Wound healing assay. (**C**) Transwell assay without matrigel. (**D**) Transwell assay with matrigel. Scale bar, 100 µm.

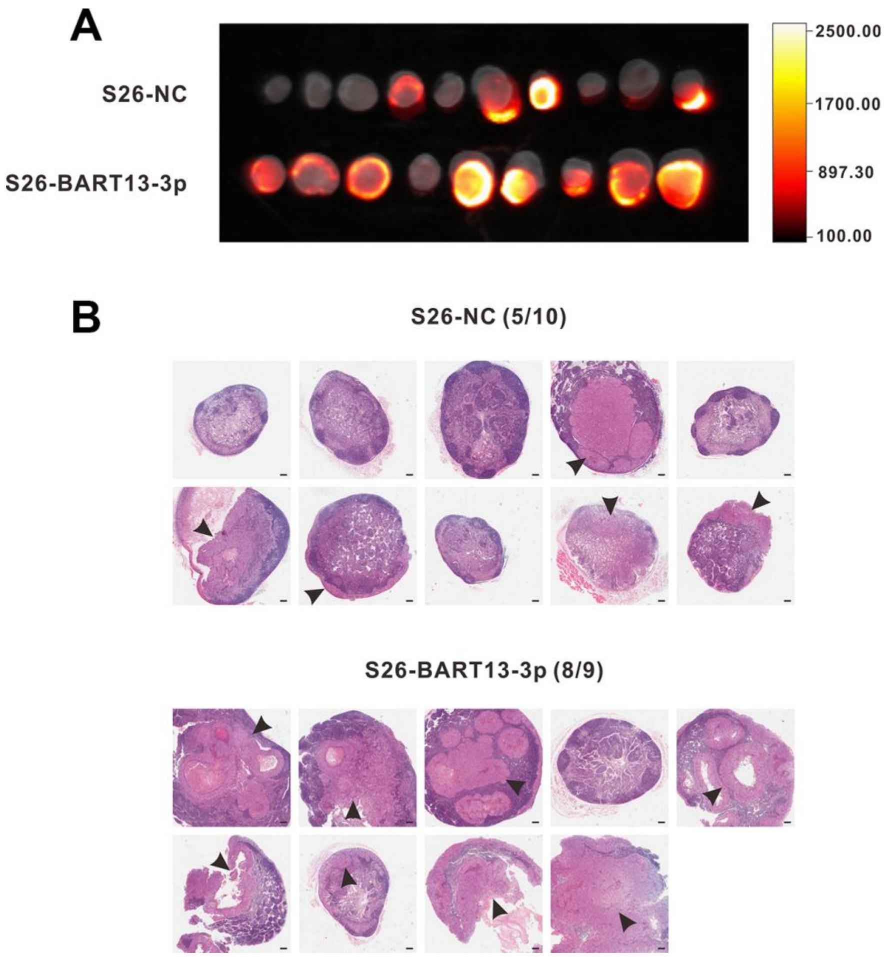

Supplementary Figure 3. EBV-miR-BART13-3p propelled NPC cells metastasis *in vivo*. (**A**) *In vivo* imaging of ipsilateral inguinal lymph nodes collected from nude mice, the foot pads of which were inoculated with S26-NC-luc or S26-BART13-3p-luc. (**B**) H&E staining of inguinal lymph nodes. Where the arrows point showed tumor metastasis. Scale bar, 200 µm.
