# Supplementary material for: Downregulation of ABI2 expression by EBV-miR-BART13-3p induces epithelial-mesenchymal transition of nasopharyngeal carcinoma cells through upregulation of c-JUN/SLUG signaling
Source: Aging (Albany NY). 2020 Jan 6;12(1):340–58. doi: 10.18632/aging.102618 (PMC6977665; doi:10.18632/aging.102618)
Supplement: Supplementary Tables [file aging-12-102618-s001..pdf]

SUPPLEMENTARY TABLES

Supplementary Table 1. Patient information.

Case n=24	Gender	Age	Cancer form	TNM stage
1	M	73	Undifferentiated cancer	T4N2M0
2	M	60	Undifferentiated cancer	T4N3M0
3	F	62	Undifferentiated cancer	T2N1M0
4	M	43	Undifferentiated cancer	T2N2M0
5	F	71	Non-keratinizing differentiated cancer	T2N1M0
6	M	70	Undifferentiated cancer	T3N1M0
7	M	61	Undifferentiated cancer	T3N1M0
8	M	28	Undifferentiated cancer	T3N3M0
9	F	55	Undifferentiated cancer	T2N1M0
10	M	61	Undifferentiated cancer	T3N3M0
11	M	43	Undifferentiated cancer	T2N1M0
12	F	52	Undifferentiated cancer	T2N0M0
13	F	54	Undifferentiated cancer	T3N3M0
14	M	47	Undifferentiated cancer	T3N2M0
15	M	77	Undifferentiated cancer	T4N1M0
16	M	27	Undifferentiated cancer	T4N2M0
17	M	60	Non-keratinizing differentiated cancer	T4N1M0
18	F	56	Undifferentiated cancer	T3N0M0
19	M	59	Non-keratinizing differentiated cancer	T4N1M1
20	M	35	Undifferentiated cancer	T2N3M0
21	M	54	Undifferentiated cancer	T2N0M0
22	M	30	Undifferentiated cancer	T4N1M0
23	M	50	Non-keratinizing differentiated cancer	T3N1M0
24	M	54	Undifferentiated cancer	T3N0M0

Supplementary Table 2. Quantitative real-time PCR primers.

miRNA	Primer	Sequence
EBV-miR-BART13-3p	miRNA	UGUAACUUGCCAGGGACGGCUGA
	RT	GTCGTATCCAGTGCAGGGTCCGAGGTATT<br>CGCACTGGATACGACTCAGCC
	Forward primer	GGGACGTGTAACTTGCCAGG
	Reversed primer	CAGTGCAGGGTCCGAGGTAT
U6	RT	CGAATTTGCGTGTCATCCT
	Forward primer	CTCGCTTCGGCAGCACATA
	Reversed primer	CGAATTTGCGTGTCATCCT
mRNA	Primer	Sequence
ABI2	Forward primer	CAAAGCCTACACCACCCAATC
	Reversed primer	AGGTTGGCTGGAGCAATAATC
c-JUN	Forward primer	TCCAAGTGCCGAAAAAGGAAG
	Reversed primer	CGAGTTCTGAGCTTTCAAGGT
SNAIL	Forward primer	TAATCCAGAGTTTACCTTCCAGC

	Reversed primer	CTCATCTGACAGGGAGGTCAG
SLUG	Forward primer	CCCTGAAGATGCATATTCGGA
	Reversed primer	CTGCAAATGCTCTGTTGCAG
CTNNB1	Forward primer	TGCTGAAGGTGCTATCTGTCTG
	Reversed primer	CCTTCCATCCCTTCCTGTTT
ZEB1	Forward primer	TCACTAGTGTTTACCAGAACAGTG
	Reversed primer	GAACACTGTTCTGGTCAGCA
ZEB2	Forward primer	CTACAAGCGCTTGACATCAC
	Reversed primer	TAGCATTTGGTGCTGATCTGTC
TWIST1	Forward primer	AGTCTTACGAGGAGCTGCAG
	Reversed primer	CTCTGGAGGACCTGGTAGAG
FOXC2	Forward primer	CTTCTACCGGGAGAACAAGC
	Reversed primer	CTCCTTCTCCTTGGACACGT
GAPDH	Forward primer	GAGTCAACGGATTTGGTCGT
	Reversed primer	GACAAGCTTCCCGTTCTCAG

Supplementary Table 3. Antibodies used for the study.

Antibody	Company	Catalog no.	Dilution ratio
**Western blotting**			
GAPDH	Servicebio	GB11002	1:2000
E-cadeherin	CST	9782T	1:1000
Vimentin	CST	9782T	1:1000
ABI2	Proteintech	14890-1-AP	1:1000
c-JUN	Abclonal	A11378	1:1000
SLUG	CST	9782T	1:1000
Rabbit	Servicebio	GB23303	1:3000
**Immunohistochemistry**			
E-cadeherin	CST	9782T	1:400
Vimentin	CST	9782T	1:200
ABI2	Proteintech	14890-1-AP	1:200
Pan-Keratin	Proteintech	26411-1-AP	1:2000
**Immunofluorescence**			
E-cadeherin	CST	9782T	1:200
Vimentin	CST	9782T	1:100
ABI2	Proteintech	14890-1-AP	1:100
c-JUN	Abclonal	A11378	1:100
SLUG	CST	9782T	1:400
